# Assessment of the availability, accessibility, and quality of sexual and reproductive health services for young people in conflict affected zones of Cameroon: a mixed method study

**DOI:** 10.1186/s12913-023-10142-1

**Published:** 2023-10-26

**Authors:** Roseline Dzekem Dine, Valentine Uwamahoro, James Olasunkanmi Oladapo, Gilbert Eshun, Fortune Benjamin Effiong, Frank Kyei-Arthur, Ayuk Bertrand Tambe

**Affiliations:** 1 Rinda Ubuzima, Kigali, Rwanda; 2 Jhpiego, Kigali, Rwanda; 3 Ladoke Akintola University of Technology Ogbomoso, Oyo State, Nigeria; 4 Seventh-Day Adventist Hospital, Agona, Asamang Ghana; 5 University of Calabar, Calabar, Nigeria; 6 University of Environment and Sustainable Development, Somanya, Ghana; 7 University of Buea, Buea, Cameroon

**Keywords:** Availability, Accessibility, Knowledge, Barriers, Sexual and Reproductive Health, Quality, Understanding, Young people

## Abstract

**Introduction:**

Despite ongoing programs to improve young people’s Sexual and Reproductive Health Services (SRHS) in the conflict plagued North West and South West Regions of Cameroon, there is limited evidence-based information evaluating SRHS. This study, therefore, aims to investigate the availability, accessibility, and quality of SRHS provided to young people in the North West and South West Regions of Cameroon.

**Method:**

This is a cross-sectional mixed-methods sequential explanatory study conducted among healthcare providers and young people between 10 and 24 years in 6 selected urban and rural areas in North West and South West regions. Data was collected between December 2021 and September 2022 using an adopted checklist. A descriptive analysis was conducted for quantitative data. An inductive analysis was conducted for the qualitative data to construct themes. The findings from the quantitative and qualitative responses were triangulated.

**Results:**

There were 114 participants, 28 healthcare providers and 86 young people. Most provider participants were nurses (n = 18, 64.3%), working in religious facilities (n = 14, 50.0%), with diplomas as state registered nurses (n = 9, 32.1%). Also, more than half of young people (51.2%) were less than 20 years old, while there were more male young people (51.2%) than female young people (48.8%). Most respondents agreed that SRHS services were available, though they think they are not designed for young people and have limited awareness campaigns about the services. Reasons such as limited use of written guidelines, affected quality of SRHS. Participants revealed shyness, resistance from religious groups and families, insecurities from political instability, and inadequate training, among others, as barriers to SRH accessibility.

**Conclusion:**

The study shows that SRHS are available but are not specifically designed for young people. Inadequate publicity for these services, coupled with the political crises and the ongoing COVID-19 pandemic, has increased young people’s inaccessibility to SRHS. Young people usually have to finance the cost of most of the SRHS. The quality of service delivery in the facilities is inadequate and must therefore be improved by developing safe, youth-friendly centers staffed with well-trained service providers.

## Background

A comprehensive Sexual and Reproductive Health, Rights, and Justice (SRHRJ) Package is needed for the sound sexual health and well-being of young people. According to the 1994 International Conference on Population and Development (ICPD), a comprehensive SRHRJ educational package should comprise an “age-appropriate, culturally relevant approach to teaching about sexuality and relationships by providing scientifically accurate, realistic, and non-judgmental information” [1–3]. Thus, for the Sexual and Reproductive Health (SRH) and well-being of young people to be achieved, a functional system that promotes sound information and services is needed.

Globally, about 410,000 young people between 10 and 24 years old were newly infected with HIV as of 2020. Among those, 150,000 were adolescents between 10 and 19 years old [4]. While the prevalence of unintended pregnancy and abortion has significantly reduced in developed countries, young people in developing countries continue to face SRHRJ challenges due to several factors, including incorrect use of contraceptives and socio-cultural norms [3, 5]. In Low-and Middle-Income Countries (LMICs), about 21 million girls between 15 and 19 years old get pregnant, and 12 million children are eventually delivered [6]. This phenomenon could be attributed to varied factors, including gender-based violence and alcohol use, lower socio-economic status, place of residence, multiple sexual partnerships, low education, and being between the ages of 20–24 [7, 8].

Sexually active adolescents between 15 and 19 years old have a higher margin (60%) of unmet needs for contraceptives. This is sometimes caused by healthcare providers’ attitudes [9], yet there are limited data on the situation among adolescents between 10 and 14 years old [1–3]. Additionally, there is a shortage of Sexual and Reproductive Health Services (SRHS) in most developing countries [10]. These pose a threat to the projected 2030 AIDS-Free Generation as there is still an increasing record of sexually transmitted infections such as HIV among young people [11–13]. For instance, in Nigeria and Rwanda, SRHS exist but are mostly present in urban areas. These services are often expensive, especially for those without health insurance to cover the cost [14, 15]. Though some young people have reported increased access to SRHS, some have complained about the complicated processes related to access, including the referral mechanisms [16]. Providers of reproductive health services in Rwanda have reported that 62.8% of their adolescent clients spend about 30 min reaching a health facility to access SRHS, and 72.2% of them indicated that they do not use social media to provide education and information on the subject to young people [15] despite increasing access to SRHS.

Even though SRHS is said to be available and accessible in most developing countries, end-users are hardly consulted on the desired quality of care they need [10]. Evidence exists that young people wish to be involved in decision-making processes concerning services that are being provided to them, but this is often not possible [17, 18]. A study conducted in Botswana to investigate the friendliness of SRHS revealed that 33% of the services were not youth-friendly, and 26% of respondents complained that healthcare providers had no respect for them despite long waiting times [16]. These indicate a need to assess SRHS provided to young people in different settings.

Cameroon has the highest rates of unmet needs for modern contraceptive methods among married (29.30%) and unmarried young people (41.70%) [19], yet each year, adolescents start sexual intercourse and childbearing. According to the Cameroon Demographic and Health Survey 2018, the prevalence of HIV is higher among young women between the ages of 15 and 24 (1.5%) compared to young men between the ages of 15 and 24 (1.1%) [20]. Young women between 15 and 24 years old are 5 to 6 times more likely to be infected with HIV/AIDS than young men, and this is similar among adolescents [21, 22]. According to the 2016–2027 health sector plan, adolescents’ needs for family planning services are unmet [22]. Currently, there are school and health facility-based programs to improve the sexual and reproductive health of young people in Cameroon [21]. Based on a 2018 forum to boost SRHS within schools and universities in Cameroon, it was found that there is a knowledge gap in the transmission of sex education [21]. Out of the 10 Regions in Cameroon, the United Nations Population Fund is actively improving SRHS in the North West and South West Regions [23] because of the weakened health system [24].

Studies in the North West region of Cameroon found that though young people use SRHS, they experience challenges in accessing them. For instance, Vifeme et al. [25] study among HIV-infected youth in the North West region of Cameroon found that most youth used SRH counselling (78%), pregnancy prevention services (70%), and STI prevention services (76%). Also, youth in the rural areas were more likely to use SRHS than those in urban areas. However, studies have found that young people in rural areas are less likely to use SRHS, such as contraceptives than those in urban areas [26, 27].

In terms of challenges in accessing SRHS, Fonkwo et al.’s [28] study in the North West region of Cameroon among community stakeholders, including the youth, found that conflict in the North West region affected the access and provisions of SRHS, and consequently, led to increased incidence of unplanned pregnancies, unsafe abortions, and sexually transmitted infections. Similarly, Haddison et al. [29] study, which compared the utilization of reproductive services before (2016–2017) and during (2018) the armed conflict in the South West region of Cameroon, found that the utilization of reproductive services (deliveries attended by skilled birth attendants, and attendance at antenatal care) deteriorated during the armed conflict compared to the before the armed conflict. Awasom-Fru et al.’s [30] study among doctors at a Catholic Hospital in the North West region of Cameroon found that adherence to Catholic rules (such as a ban on advice and prescription of modern contraceptives within the hospital) and perception of fear within the hospital environment limited the accessibility of SRHS to hospital clients, including adolescents.

Though there are ongoing programs to improve young people’s SRHS in the North West and South West Regions of Cameroon, there is limited evidence-based information evaluating the SRHS situation in these areas. Therefore, this study seeks to assess the availability, knowledge, understanding, accessibility, and quality of SRHS provided to young people within the North West and South West Regions of Cameroon towards the promotion of health and well-being of adolescents and young people.

## Methods

### Study design and sampling procedure

This study used a cross-sectional mixed-methods sequential explanatory design to assess the availability, knowledge, understanding, accessibility, and quality of SRHS provided to young people within the North West and South West Regions of Cameroon. A mixed-method sequential explanatory design is a type of mixed-method design where qualitative data collection and analysis come after quantitative data collection and analysis [31]. The qualitative findings help to further explain the quantitative findings. In this study, a survey was used to collect the quantitative data because it is easy to use and captures the current sexual and reproductive health situation for young people within the selected cities of Cameroon. Young people aged 10–24 were conveniently selected from the North West and South West regions. In each region, three towns were selected. In each selected city, between 9 and 37 young people were selected. Also, health facilities providing SRHS were conveniently selected, and volunteering providers were recruited to be a part of this study from the selected cities. In each region, 3 health facilities were chosen conveniently, and within each health facility, between 3 and 5 providers were participants.

### Study setting

Cameroon has 10 regions, of which the North West and South West, among others, are humanitarian settings. These two regions are commonly known as the English speaking parts of Cameroon. Our study was conducted in selected urban and rural areas of these regions. In the North West region, the study was conducted in Bambui, Bambili, and Bamenda, while in the South West region, the study was conducted in Limbe, Mutengene, and Buea. The urban areas were Bamenda, Limbe and Buea, while Bambui, Bambili and Mutengene were rural. Young people in these regions face increased reproductive health challenges. Several humanitarian activities to better the lives of the citizens, as well as some programs on reproductive health to promote young people’s health, are ongoing.

### Study population

We used convenient and snowballing sampling techniques to recruit 114 participants for this study. Twenty-eight [32] healthcare providers (nurses, social workers, and other SRHS providers) were selected and interviewed because of their ability to provide information. Using the snowball sampling method, 86 young people between 10 and 24 years of age from the selected cities were recruited to participate in this study. Snowballing was employed to reach out to young people because of the instability within the selected study site. All participants were residents or practitioners in one of the 6 selected cities and had the ability to communicate in English effectively.

### Measurement of variables

Knowledge of SRHS was measured by the availability of SRHS. Young people and healthcare providers were asked to respond “yes” or “no” to the availability of several SRHS, including HIV testing, STI testing, contraceptive methods, pregnancy tests, and antenatal and postnatal care, among others.

The accessibility of SRHS was measured by asking young people and healthcare providers to respond “yes” or “no” to access to SRHS via social media, suitable operational hours, access to medical records, location from residence to facility, waiting time, and service cost.

The quality of SRHS was measured by asking young people and healthcare providers to respond “yes” or “no” to the use of written guidelines, staff demonstrate respect when interacting with young people, privacy in services provision, the privacy of the rooms, comfortable waiting area, peer counselors, sound feedback mechanism, and needs of Adolescents satisfied.

### Data collection

This study was conducted between December 2021 and September 2022 among young people and healthcare providers in charge of SRHS in the selected centers. Young people were recruited from the community, while health providers came from the health facilities where they practiced. A validated questionnaire by the World Health Organization and Pathfinder International was adapted for this study [4, 33]. The questionnaire was partitioned into sections (availability, knowledge, understanding, accessibility, quality, and barriers). Four (4) research assistants were recruited from the Universities of Buea and Bamenda in Cameroon to assist in the data collection. Each interview took an average of an hour, including signing consent or assent forms.

### Ethical considerations

#### Ethical approval

Ethical approval for this study was obtained from the Institutional Review Board, Faculty of Health Sciences, University of Buea, Cameroon. Administrative authorization for data collection was sought from the South West and North West Regional Delegations of Public Health. The study was implemented according to Helsinki’s declaration of human subject research. The child’s parents or guardians were given a written consent form and discussed it with a study data collector. They were given ample time to ask questions before deciding whether to allow their children to participate. This applied to all participants. If participants agreed to participate, they signed an informed consent/assent form or provided their thumbprint, witnessed by a non-study staff member. All identifiable information was kept confidential. They could be saved in a locked cupboard or a passworded computer.

### Data analysis

Quantitative data was entered into Microsoft Excel, cleaned, and exported to SPSS version 26.0 for analysis. Descriptive statistics, such as frequencies and percentages, were used to describe the characteristics of respondents and the availability, knowledge, understanding, accessibility, and quality of SRHS provided to young people.

ATLAS Ti version 5.1 was used to analyze qualitative data. Using an inductive approach, a codebook was initially developed to summarize data. The codebook was enriched as the data collection process went on. All information obtained from participants during the interviews was later coded separately from their personal information. Themes were generated and enriched by sub-themes and explanations (Table 1).

**Table 1:** Thematic framework

Themes	Sub-themes	Codes	Definition
Availability	Services available in health facilities or communities for young people	Services available	We defined it as the presence of services for young people to use.
		Services not available	
Knowledge	Knowledge of the availability of SRHS	Having knowledge	It was defined as young people and providers’ ability to know that the services are available somewhere for young people to improve their sexual and reproductive health.
		Not having knowledge	
Understanding	Understanding how SRHS are provided	Positive understanding	We defined this as how young people and service providers perceive what should be included in the SRHS package.
		Negative understanding	
Accessibility	The act of being able to access services	Services are accessible.	We measured the user-friendliness of these services to young people in terms of access.
		Services are not accessible	
Quality	Judgment given to the nature of the services and resources	Of quality	Here, we looked at how well the services are provided or meeting the needs of young people.
		Not of quality	
Barriers	Barriers towards accessing or utilizing SRHS	Existence of a barrier	These are those setbacks that young people face while willing to access sexual and reproductive health services.

## Results

These results are presented according to our aim, namely: the availability, knowledge, understanding, accessibility, and quality of sexual and reproductive health services provided to young people within the North West and South West Regions of Cameroon, in addition to the description of study participants and possible barriers to accessing services.

### Socio-demographic characteristics of healthcare providers and young people

Table 2 shows the socio-demographic characteristics of 28 healthcare providers interviewed. Half (50.0%) of healthcare providers were affiliated with religious facilities, while 46.4% were affiliated with the government. A higher proportion (17.9%) of healthcare providers were located in Bambili, Bambui, Buea, and Mutengene, respectively. In addition, the majority (64.3%) of healthcare providers were nurses. Most healthcare providers had obtained a diploma as state registered nurses (32.1%).

**Table 2:** Socio-demographic characteristics of health providers in South West & North West of Cameroon (n = 28)

	Frequency	Percentage
**Location of the health facility**		
Bambili	5	17.9
Bambui	4	14.2
Bamenda	4	14.2
Buea	5	17.9
Limbe	5	17.9
Mutengene	5	17.9
**Affiliation of health providers**		
Government	13	46.4
Private	1	3.6
Religious	14	50.0
**Job title**		
Psycho-social counselor	1	3.6
Nurse	18	64.3
Midwife	5	17.8
Peer educator	1	3.6
Student	2	7.1
Community health	1	• 3.6
**Educational qualification**		
Advanced level	1	3.6
Bachelor in international development	1	3.6
Bachelor in international relationship and conflict resolution	1	3.6
Bachelor in midwifery	5	17.8
Bachelor in nursing	1	3.6
Higher national diploma in nursing	8	28.5
Master in public health	1	3.6
State diploma in midwifery	1	3.6
Diploma in state registered nursing	9	32.1

Table 3 presents the socio-demographic characteristics of 86 young people interviewed. Less than one-third of young people (31.4%) were less than 18 years old, while there were more male young people (51.2%) than female young people (48.8%). Also, most young people were from Bambili (26.7%) and had tertiary education (44.2%).

**Table 3:** Socio-demographic characteristics of young people in South West & North West of Cameroon (n = 86)

	Frequency	Percentage
**Age of participants (years)**		
10–13	8	9.3
14–17	19	22.1
18–21	27	31.4
22+	32	37.2
**Sex**		
Female	42	48.8
Male	44	51.2
**Educational level of participants**		
No formal education	6	7.0
Primary level	7	8.1
Secondary (Ordinary/Advanced level)	35	40.7
Tertiary	38	44.2
**Location**		
Bambili	23	26.7
Bamenda	12	14.0
Bambui	12	14.0
Buea	13	15.1
Limbe	13	15.1
Mutengene	13	15.1

### Availability of Sexual and Reproductive Health Services for Young People

Table 4 depicts the availability of sexual and reproductive health services for young people. The finding shows that the majority (82.9%) of the young people who participated in the survey had access to HIV testing services, with 78.6% of the service providers also agreeing to provide such services. More than half of young people (64.1%) indicated that HIV self-testing kits were not readily available at the facilities, though 67.9% of the service providers reported that they were readily available. Likewise, 61.3% of the young people also reported that initiation of HIV therapy on the same day of diagnosis was unavailable. However, 78.6% of healthcare providers reported they were available for young people at various facilities. In addition, 8 out of 10 young people (83.8%) reported that HIV counseling services are available at the facilities, which 85.7% of service providers attest to.

**Table 4:** Availability of Sexual and Reproductive Health Services for young people

Variables	Young people			Providers		
	Total	YES n(%)	NO n(%)	Total	YES n(%)	NO n(%)
HIV testing	82	68(82.9)	14(17.1)	28	22(78.6)	6(21.4)
STIs testing	78	55(70.5)	23(29.5)	28	21(75)	7(25)
HIV self-testing kits	78	28(35.9)	50(64.1)	28	19(67.9)	9(32.1)
STIs treatment	74	35(47.3)	39(52.7)	28	22(78.6)	6(21.4)
HIV same-day therapy initiation	75	29(38.7)	46(61.3)	28	22(78.6)	6(21.4)
HIV counseling	80	67(83.8)	13(16.2)	28	24(85.7)	4(14.3)
Contraceptive methods	75	48(64.0)	27(36.0)	27	27(100)	*
Pregnancy test	72	50(69.4)	22(30.6)	*	*	*
Intrauterine device (IUD)	69	28(40.6)	41(59.4)	*	*	*
Implant	70	32(45.7)	38(54.3)	*	*	*
Combined oral contraceptives	70	31(44.3)	39(55.7)	27	27(100)	*
Progesterone contraceptives	69	30(43.5)	39(56.5)	27	27(100)	*
Emergency contraceptives	69	27(39.1)	42(60.9)	27	27(100)	*
Depo Provera injection	70	32(45.7)	38(54.3)	27	27(100)	*
Male condoms	71	51(71.8)	20(28.2)	27	27(100)	*
Female condoms	69	36(52.2)	33(47.8)	27	27(100)	*
Vasectomy	67	19(28.4)	48(71.6)	*	*	*
Tubal ligation	68	32(47.1)	36(52.9)	*	*	*
Circumcision	68	42(61.8)	26(38.2)	*	*	*
Lubricants	65	26(40.0)	39(60.0)	*	*	*
Fertility awareness	69	36(52.2)	33(47.8)	*	*	*
Antenatal care (ANC)	68	41(60.3)	27(39.7)	28	8(28.6)	20(71.4)
Post-natal care	68	41(60.3)	27(39.7)	28	7(25)	21(75)

*: No responses

Regarding the availability of services for testing other STIs (excluding HIV), most young people (70.5%) and service providers (75%) revealed that the services are available. In contrast, 47.3% of the young people agreed that STI treatment was available, while 78.6% of the service providers agreed that services for STI treatment were readily available.

Young people indicated that the following sexual and reproductive health services were available: pregnancy testing (69.4%), contraception (64.0%), circumcision (61.8%), fertility awareness (52.2%), antenatal care (60.3%), and postnatal care (60.3%). Of the contraceptive methods, young people indicated that male condoms (71.8%), female condoms (52.2%), depo provera (45.7%), progesterone-only pills (43.5%), implants (45.7%), combined oral contraceptives (44.3%), lubricants (40.0%), intrauterine devices (40.6%), emergency contraceptives (39.1%), tubal ligation (47.1%), and vasectomy (28.4%) were available. Young people from Bambui (11.1%) experience limited availability of SRHS (Table 5).

**Table 5:** Location of participants by availability of sexual and reproductive health services

Location	Availability of Sexual and Reproductive Health Services	
	Frequency	Percentage
Bambili	22	27.3
Bambui	9	11.1
Bamenda	13	16.0
Buea	12	14.8
Limbe	12	14.8
Mutengene	13	16.0
Total	81	100.0

All service providers indicated that contraception methods (excluding female condoms) were available in the various facilities. Our findings also revealed that most SRHS for young people are available but not mainly there for them since most facilities were constructed for the general public and thus might not have comprehensive sexual and reproductive health packages for young people.

*“There are supplies available onsite (medical testing, treatment, amongst others), readily available.” (*Provider_Bambili*)*.

*“Not all especially specialized services” (*Provider_Mutengene*); “It depends, but not for everyone” (*Young person_Bamenda*)*.

It was also found that, generally, condoms were available, though few said that female condoms were mostly not available for young people.

“*Only for female condoms are not available*” (Young person_Mutengene).

### Young people and provider’s knowledge of sexual and Reproductive Health services

In Tables 6, 25.3% of young people revealed that information is regularly disseminated to raise awareness about the availability of reproductive health services and sites. In comparison, 26.6% reported that it’s not being done at all. On the other hand, 65.4% of providers reported that they do not raise awareness of SRHS.

**Table 6:** Young people and provider’s knowledge of Sexual and Reproductive Health Services

Variables	Young people			Providers	
	Total	Frequency n(%)		Total	Frequency
**Information spread on services (Campaigns/awareness)**					
*Always*	79	20(25.3)	Yes	9	34.6
*Sometimes*	79	38(48.1)	No	17	65.4
*Never*	79	21(26.6)			*

*: *No response*

The qualitative findings demonstrated that young people are poorly informed on sexual and reproductive health services and their uses. Most young people affirmed that they sometimes hear publicities about SRHS. At the same time, the service providers reported that they spread information about the presence, availability, and accessibility of SRHS via television, radio, home visits, and megaphones.

*“I know that the facility avails information to the public on SRHS provision via the Radio* (provider_Bamenda), “*before starting to provide services, we conduct a counseling session to prepare clients and also get them abreast of the package”* (provider_Bambili), “*We use mass media tools such as TV and use of town crier to raise awareness”* (provider_Buea), as well as “*home visits at least for instance 5 times in a year*” (provider_Limbe).

### Providers and young people’s understanding of sexual and Reproductive Health services

As shown in Table 7, the majority of respondents (86.3% and 92.9% of the young people and service providers, respectively) expressed that health facilities provided more information on SRHS when requested. In addition, most young people (81.3%) mentioned that health facilities provided more information about general health than specific information about sexual and reproductive health. Most respondents (57.1%) indicated that there was enough time for interaction in various health facilities between clients and service providers. More than half of young people (50.7%) reported that there was transparency in the services of service providers, while most service providers (77.8%) explained that they were transparent in the services provided.

**Table 7:** Providers and young people understanding of Sexual and Reproductive Health Services

Variables	Young people			Providers		
	Total	YES n(%)	NO n(%)	Total	YES n(%)	NO n(%)
More information availed on services provided	80	69(86.3)	11(13.7)	28	26(92.9)	2(7.1)
More information on general health	80	65(81.3)	15(18.7)	28	28(100)	*
Time for interaction	77	44(57.1)	33(42.9)	28	22(78.6)	6(21.4)
Transparent providers	69	35(50.7)	34(49.3)	27	21(77.8)	6(22.2)

*: *No response*

Service providers and young people had a different understanding of the contact hours and periods for the provision of SRHS. According to service providers, these services are meant to be provided mostly during Cameroon working hours (7 am GMT + 1 to 2 pm GMT + 1) or during special events such as HIV special meeting days (Saturdays) for young people who were affiliated with treatment centers.

“*Everything we do, including the provision of SRHS, is done within working hours, and the closing time remains the same. It’s an inclusive task without special considerations*” (Health provider_Buea).

Some young people do not have enough knowledge and understanding of the purpose and content of SRHS.

“*No idea at all because I am not accessing the SRHS*” (Young person_Buea);

“*Never, I have not heard of such*” (young person_Limbe).

“*Never, and even if I have heard any information about SRH, it has only been at school, not within the community. Our teacher told us it works in class, and sometimes it just comes up because of the subject being taught, often Biology or Human Biology*” (Young person_Limbe).

### Young people and providers’ perception of the accessibility of sexual and Reproductive Health services

The quantitative and qualitative data findings revealed that social media (Facebook, WhatsApp, and Twitter, amongst others) channels are somehow being used to provide SRHS. Young people in Bamenda (4.0%) experienced difficulty in accessing SRHS (Table 8).

**Table 8:** Location of participations by accessibility of sexual and reproductive health services

Location	Variables on accessibility of Sexual and Reproductive Health Services	
	Frequency	Percentage
Bambili	20	39.2
Bambui	8	15.7
Bamenda	2	4.0
Buea	7	13.7
Limbe	7	13.7
Mutengene	7	13.7
Total	51	100.0

Table 9 indicates that about 49.4% and 60.0% of young people and service providers, respectively, reported the use of social media in the provision of SRHS. According to service providers, health facilities are very accessible. Specifically, 92.6% of service providers reported that health facilities were less than 30 min away from most young people’s place of residence. However, less than half of young people (44.5%) mentioned that health facilities were less than 30 min apart from their place of residence. Most respondents (60.9% and 70.4% of the young people and the providers respectively) agreed they have access to their medical records. Generally, most young people from Bambili had their residences far from facilities to access SRHS (Table 10). Similarly, most young people from Bambili had to wait longer to access SRHS (Table 11). Most young people from Bambili (28.6%) and Limbe (28.6%) perceived the service cost of SRHS as high (Table 12).

**Table 9:** Young people and providers’ perception of the accessibility of Sexual and Reproductive Health Services

Variables	Young people			Providers		
	Total	YES n(%)	NO n(%)	Total	YES n(%)	NO n(%)
Social media usage	79	39(49.4)	40(50.6)	25	16(64)	9(36)
Suitable operational hours	72	46(63.9)	26(36.1)	27	15(55.6)	12(44.4)
Access to medical records	69	42(60.9)	27(39.1)	27	19(70.4)	8(29.6)
**Accessibility in terms of location from residence to facility**						
*Accessibility to location (Less than 30 min)*	81	36(44.5)	*	27	25(92.6)	2(7.4)
*Accessibility in location (30 min*^*− 1*^ *h)*	81	27(33.3)	*	*	*	*
*Accessibility in location (1 h and above)*	81	18(22.2)	*	*	*	*
**Waiting time**						
*Less than 30 min*	70	37(52.9)	*	23	18(78.3)	5(21.7)
*31–60 min*	70	19(27.1)	*	*	*	*
*More than 1 h*	70	14(20.0)	*	*	*	*
**Service cost**						
*Free*	72	12(16.7)	*	27	24(88.9)	3(10.7)
*Low cost*	72	40(55.6)	*	27	24(88.9)	3(10.7)
*High cost*	72	20(27.7)	*	*	*	*

*: *No response*

**Table 10:** Location of participations by of location from residence to facility

	Accessibility in terms of location from residence to facility		
	*Less than 30 min*	*31–60 min*	*More than 1 h*
**Location**	**n(%)**	**n(%)**	**n(%)**
Bambili	4 (16.7)	11 (64.7)	4 (33.3)
Bambui	0 (0.0)	2 (11.8)	4 (33.3)
Bamenda	0 (0.0)	0 (0.0)	0 (0.0)
Buea	6 (25.0)	0 (0.0)	3 (25.0)
Limbe	7 (29.2)	1 (5.9)	1 (8.3)
Mutengene	7 (29.2)	3 (17.6)	0 (0.0)
Total	24 (100.0)	17 (100.0)	12 (100.0)

**Table 11:** Location of participations by waiting time

	Waiting time		
	*Less than 30 min*	*31–60 min*	*More than 1 h*
**Location**	**n(%)**	**n(%)**	**n(%)**
Bambili	9 (27.3)	4 (66.7)	7 (38.9)
Bambui	3 (9.1)	1 (16.7)	3 (16.7)
Bamenda	7 (21.2)	0 (0.0)	0 (0.0)
Buea	4 (12.1)	0 (0.0)	3 (16.7)
Limbe	7 (21.2)	0 (0.0)	2 (11.1)
Mutengene	3 (9.1)	1 (16.7)	3 (16.7)
Total	33 (100.0)	6 (100.0)	18 (100.0)

**Table 12:** Location of participations by service cost

	Service cost		
	*Free*	*Low cost*	*High cost*
**Location**	**n(%)**	**n(%)**	**n(%)**
Bambili	7 (31.8)	10 (41.7)	4 (28.6)
Bambui	2 (9.1)	2 (8.3)	2 (14.3)
Bamenda	7 (31.8)	0 (0.0)	0 (0.0)
Buea	2 (9.1)	3 (12.5)	2 (14.3)
Limbe	1 (4.5)	5 (20.8)	4 (28.6)
Mutengene	3 (13.6)	4 (16.7)	2 (14.3)
Total	22 (100.0)	24 (100.0)	14 (100.0)

Regarding cost, young people and service providers mentioned that some of the services are free, while some are provided at a low or high cost, such as the HIV testing services provided in most, if not all, public facilities at zero cost.

“*We use social media tools such as Facebook to gain knowledge of SRH, but not part of the facility from which we request for treatment*” (Young person_Bambili).

“*Yes, but some of them live far away yet manage to come, some, we pay their transport supported by Cameroon Baptize Convention and Catholic Relief Fund*” (Provider_Limbe).

“*A youth-friendly clinic is needed. Operational hours are suitable for young people but no particular clinic for them*” (Provider_Mutengene).

### Providers’ and young people’s perceptions about the quality of sexual and Reproductive Health services

In Tables 13, 69.4% of young people said their needs are satisfied when they attend SHRS. Young people and service providers agreed that writing guidelines are used in service delivery (89.4% and 70.4%, respectively), and staff shows respect when interacting with clients (77.0% and 96%, respectively). They also agreed that staff respect privacy when delivering the services (78.4% and 92.9%, respectively) and that the rooms used when delivering the services allow for privacy (70.3% and 96.2%, respectively). In addition, the majority of the young people and providers (64.9% and 76.9%, respectively) revealed that peer counselors are used in the facilities. Nevertheless, the majority (68.9%) of the young people responded that there is no sound feedback mechanism between clients and service providers, though 61.5% of providers said otherwise.

**Table 13:** Providers’ and young people’s perceptions about the quality of Sexual and Reproductive Health Services

Variables	Young people			Providers		
	Total	YES n(%)	NO n(%)	Total	YES n(%)	NO n(%)
Use of written guideline	66	59(89.4)	7(10.6)	27	19(70.4)	8(29.6)
Staff demonstrate respect when interacting with young people	74	57(77.0)	17(23.0)	25	24(96)	1(4)
Privacy in services provision	74	58(78.4)	16(21.6)	28	26(92.9)	2(7.1)
Privacy of the rooms	74	52(70.3)	22(29.7)	26	25(96.2)	1(3.8)
Comfortable waiting area	77	58(75.3)	19(24.7)	27	26(96.3)	1(3.6)
Peer counselors	77	50(64.9)	27(35.1)	26	20(76.9)	6(23.1)
Sound feedback mechanism	74	23(31.1)	51(68.9)	26	16(61.5)	10(38.5)
Needs of Adolescents satisfied (meeting needs)	72	50(69.4)	22(30.6)	*	*	*

*: *No response*

During the interviews, service providers expressed that young people are not involved in designing the materials used in facilities. The respondents think that they need additional training to understand better, respond, and serve the needs of young people. Again, service providers and young people complained that the space for SRHS is small. Furthermore, young people do not appreciate the current method used to record their medical history as they feel it is unsafe to use the analog system.

“We receive g*eneral training at school that lasts between 2 and 3 years before starting our practice within facilities. We aren’t trained specifically on SRH, but we will appreciate being trained as it would help us improve the health of our young people*” (Provider_Limbe);

“*Because of insufficient space, some people are being forced to stand while seeking services, but we try to provide appropriate services*” (Provider _Bambui).

“*We use books for patient records, and in case these books get missing, it’s hard to find patient information because we do not do electronic medical records. Several constraints such as power failure and internet connection might not support new technology in our facilities*” (Young person_Buea).

### Young people and service providers’ perspectives on barriers to accessing SRHS

In Table 14, most young people revealed that only at 14–16 (30.4%) and 17–19 (30.4%) should a young person start accessing SRHS.

**Table 14:** Young people and providers reported barriers to access Sexual and Reproductive Health Services

Variables	Young people			Providers		
	Total	Frequency (%)		Total	Frequency	
**Acceptable age to access SRHS**						
*10–13*	*79*	*19(24.1)*		*	*	
*14–16*	*79*	*24(30.4)*		*	*	
*17–19*	*79*	*24(30.4)*		*	*	
*20+*	*79*	*12(15.1)*		*	*	
**Presence of any other form of barrier**	**Total**	**YES n(%)**	**NO n(%)**	**Total**	**YES n(%)**	**NO n(%)**
	*	*	*	28	23(82.1)	5(17.9)

*: *No response*

Participants also mentioned several barriers, such as being shy, lack of knowledge in accessing SHRS, resistance from religious groups and families, insecurities, and inadequate training, to be the leading factors for not accessing these services.

“*Contraception such as condoms has resistance from some religious groups and some families*” (Provider_Mutengene);

“*Insecurity when coming to work, specifically on Mondays. It is not easy because they are threatening.* (Provider_Limbe)

“*Religious leaders: most religious institutions have various beliefs which turn to serve as a limitation to some of these services, for example, the Muslim and Catholics*” (Young person_Bambui).

## Discussion

This study investigated the availability, accessibility, and quality of SRHS provided to young people among 114 respondents within the North West and South West regions of Cameroon. Our findings showed that the overall quality of services provided to young people in these regions of Cameroon are not up to recommended standards. This is despite the availability of services to a certain extent in most facilities. Factors such as limited effective community mobilization, sensitization, and environmental factors contribute significantly to the general quality of SRHS. Young people and providers mentioned that being ashamed, fear, and insecurity significantly affect their access to SRHS. This is despite the fact that optimum utilization of these services is vital to help end the spread of some infectious diseases such as HIV/AIDS and sexually transmitted infections. These findings are similar to previous studies, which identified fear or embarrassment, lack of confidentiality and privacy as barriers to young people’s use of SRHS [34–38].

Current findings demonstrated the availability of SRHS for young people at different health facilities in the South West and North West Regions of Cameroon, but not designed specifically for young people. In Cameroon, each health level has an essential health package made available to the general population. This probably explains why the structure of SRHS has been reported to be designed for adults. These findings are similar to studies in Rwanda [15] and Ghana [39], which found that SRHS were available but not necessarily designed for young people. This situation negatively impacts young people’s utilization of SRHS.

Many of the young people in our study had limited knowledge of the availability, accessibility, and usage of SRHS at various health facilities. This might be because there are limited community mobilization and engagement activities to increase knowledge and access to SRHS. A study conducted in Tanzania also had similar findings about poor publicity and low awareness about SRHS [40]. This causes adolescents to underutilize SHRS [32]. This probably might explain the growing rates of teenage and/or unplanned pregnancies among young people, especially amid the natural and political pandemics [41]. In addition, several findings have revealed that taking extra measures to create awareness and provide interventions greatly contributes to young people cultivating sound SRH behaviors. For instance, in Ghana, it has been found that parents and/or teachers find it difficult to provide young people with SRH information [42]. However, they believe trained personnel, such as psychologists, should be able to provide SRH information.

Additionally, similar to other studies [43, 44], our study revealed that some service providers and young people have an insufficient understanding of what constitutes young people’s SRHS. There are limited avenues to promote SRHS, such as training centers to provide opportunities for young people to enhance their understanding and knowledge of SRH. Similarly, other authors reported that young people in their study did not know what and how to use SRH tools, in addition to existing myths, including using certain pharmaceutical products (decaris) that will prevent pregnancy [45–48]. Another study found that unskilled service providers pose a serious setback, affecting young people’s understanding and knowledge of SRH [49]. The lack of information, knowledge, and understanding of SHR leads to misconceptions. For instance, in Saudi Arabia, young people held negative views towards sex because they rarely received SRH information from their parents or teachers and preferred the internet as their source of information [50].

In this study, accessibility was comparatively low. There was no specialized adolescent/youth SHR clinic/center and limited or no use of social media to provide SRHS. These centers have been appreciated in different settings, such as Zambia, where young people preferred accessing services via youth health centers located in the community rather than the health facility [49]. A study also found that youth-friendlier SHRS are achieved when service providers are trained, and youth centers or corners are created for the youth to interact freely with peers and providers about their sexual and reproductive issues [32].

Both young people and providers affirmed that the conflict crises, including pandemics in the regions, have drastically affected young people’s access to health services. Like in South Africa, the lack of access to biomedical interventions (for instance, contraception) increased with the outbreak of the COVID-19 pandemic. This lack of access in South Africa caused an increase in young people’s pregnancies, gender-based violence, and sexual activities [47]. Similarly, Fonkwo et al. [28] and Haddison et al. [29] found that conflicts in the North West and South West regions of Cameroon affected access and provisions of SRHS.

We found service quality to be substandard. For instance, the cost of services depended on the sort of services that were being requested. This is despite the fact that most young people are dependent and the fact that most citizens are uninsured, as only 1% of the Cameroonian population are insured [51]. This issue of cost has also been highlighted in Ghana, where authors appreciate the point to inform young people about SRHS but fear they might not have the finances to cater for the cost [42, 45]. Other studies have also found that cost was a considerable barrier to accessing and utilizing SRHS [52]. Thus, the cost of quality services may hinder young people’s patronage of these services.

Our findings also revealed that young people are not included in the design of SRHS, though it has been found that engaging them in the design has a positive role in effectiveness [49]. Our study found that privacy was ensured in service delivery, which is in contrast to Mchome et al. [53] findings, where there was a lack of confidentiality and privacy in service delivery. This pushed adolescents to sort for local drug shops and traditional healers because they provide maximum privacy and confidentiality [54]. Health providers’ attitude also influences young people’s access and utilization of SHRS since this determines adolescents’ decisions [35, 55]. Our study found a positive attitude among service providers, though some degree of rudeness was observed.

Furthermore, we realized that access to SRHS is not always to the maximum because of different types of barriers in the communities. Like in Cameroon, it has been found elsewhere that existing barriers disturb the utilization and access to SRHS in several settings [56]. Some key barriers to access include shame and stigma and the judgmental attitudes of health providers [34–38, 45, 57, 58].

### Strengths, limitations, and prospective

We believe results from this study have limited bias because perspectives on services were obtained from both service providers and adolescents. Another strength of this study is the use of mixed methods (both qualitative and quantitative methods). This most likely helped enhance the study findings. A limitation of this study is that it is a cross-sectional study involving participants from conveniently selected sites. This means the results from the study cannot be generalized to the whole population of service providers and adolescents in Cameroon. Also, public hospitals in Cameroon provide varied packages of SHRS. Therefore, conveniently selecting public health hospitals may influence the findings on the views of healthcare providers and the different packages of SHRS provided to young people. In addition, the measurement of knowledge of SRHS by the availability of SRHS is not a robust measure of knowledge of SRHS.

Furthermore, most healthcare providers (64.3%) interviewed were nurses, which may bias the findings on healthcare providers’ perspectives on the availability, accessibility, and quality of SRHS for young people. Other limitations concern the lack of accountability of the participants’ sexual orientation and gender identity, including being transgender, as this might affect the accessibility and quality of SHRS rendered to them. As a recommendation, a well-planned program that increases awareness should be considered to ensure the availability of services are enjoyed by the targeted end users. Also, youth-friendly centers should cultivate the culture of involving young people in the design of SRHS to capture their voices. In addition, there must be a periodic evaluation of SRHS offered to ensure their relevance and attainment of the intended goals. Further studies should be done on this subject among the youth, taking into consideration their sexual orientation.

## Conclusion

The study demonstrated that SRHS are available to young people but are not specifically designed for them. Inadequate publicity of these services, coupled with the crises in these regions, has increased the challenges of young people. Social media, which is now a channel to reach young people, has not been fully utilized in the South West and North West regions of Cameroon, yet has financial access problems despite the low insurance coverage in the country. The quality of service delivered in the facilities is not the best and must therefore be improved. Thus, establishing secure, youth-friendly facilities with trained service providers who are aware of young people’s sexual and reproductive health needs will help achieve universal goals.

## Data Availability

The datasets generated and/or analyzed during the current study are not publicly available for ethical purposes but are available from the corresponding author on reasonable request.

## Abbreviations

HIV/AIDS: Human Immunodeficiency Virus/Acquired Immunodeficiency Syndrome
SRHRJ: Sexual and Reproductive Health, Rights, and Justice
ICPD: International Conference on Population and Development
LMICs: Low-and Middle-Income Countries
STIs: Sexually Transmitted Infectious
SRHS: Sexual and Reproductive Health Services
COVID-19: Coronavirus
SRH: Sexual and Reproductive Health
